# *In Vivo* Prevention of Implant-Associated Infections Caused by Antibiotic-Resistant Bacteria through Biofunctionalization of Additively Manufactured Porous Titanium

**DOI:** 10.3390/jfb14100520

**Published:** 2023-10-16

**Authors:** Ingmar Aeneas Jan van Hengel, Bruce van Dijk, Khashayar Modaresifar, Johan Frederik Felix Hooning van Duyvenbode, Faisal Ruben Hamzah Aziz Nurmohamed, Marius Alexander Leeflang, Adriaan Camille Fluit, Lidy Elena Fratila-Apachitei, Iulian Apachitei, Harrie Weinans, Amir Abbas Zadpoor

**Affiliations:** 1 Department of Biomechanical Engineering, Faculty of Mechanical, Maritime, and Materials Engineering, Delft University of Technology, Mekelweg 2, 2628 CD Delft, The Netherlandsi.apachitei@tudelft.nl (I.A.); h.h.weinans@umcutrecht.nl (H.W.); a.a.zadpoor@tudelft.nl (A.A.Z.); 2 Department of Orthopedics, University Medical Center Utrecht, 3584 CX Utrecht, The Netherlands; 3 Department of Medical Microbiology, University Medical Center Utrecht, 3584 CX Utrecht, The Netherlands

**Keywords:** additive manufacturing, titanium bone implants, surface biofunctionalization, MRSA, implant-associated infection, bone infection model

## Abstract

Additively manufactured (AM) porous titanium implants may have an increased risk of implant-associated infection (IAI) due to their huge internal surfaces. However, the same surface, when biofunctionalized, can be used to prevent IAI. Here, we used a rat implant infection model to evaluate the biocompatibility and infection prevention performance of AM porous titanium against bioluminescent methicillin-resistant *Staphylococcus aureus* (MRSA). The specimens were biofunctionalized with Ag nanoparticles (NPs) using plasma electrolytic oxidation (PEO). Infection was initiated using either intramedullary injection *in vivo* or with *in vitro* inoculation of the implant prior to implantation. Nontreated (NT) implants were compared with PEO-treated implants with Ag NPs (PT-Ag), without Ag NPs (PT) and infection without an implant. After 7 days, the bacterial load and bone morphological changes were evaluated. When infection was initiated through *in vivo* injection, the presence of the implant did not enhance the infection, indicating that this technique may not assess the prevention but rather the treatment of IAIs. Following *in vitro* inoculation, the bacterial load on the implant and in the peri-implant bony tissue was reduced by over 90% for the PT-Ag implants compared to the PT and NT implants. All infected groups had enhanced osteomyelitis scores compared to the noninfected controls.

## 1. Introduction

Implant-associated infections (IAIs) are a devastating complication for patients who undergo total joint replacement, trauma or bone tumor resection surgeries [1,2,3]. These infections also form significant social and financial burdens for society in general and healthcare systems in particular. As the number of implants continues to increase [4], the urgency of addressing IAIs further grows. Moreover, an increasing incidence of antibiotic-resistant bacteria [5] has only intensified this urgency. Given that the treatment of IAIs, particularly those caused by multidrug-resistant bacteria, is associated with extremely high human and monetary costs, the focus has shifted to the prevention of IAIs via the synthesis of self-defending implants [6,7].

While infection prevention is important for all types of orthopedic implants, it is even more so for additively manufactured (AM) porous implants, which are increasingly being used in clinical settings because of their favorable mechanical properties [8,9] and high potential for bony ingrowth [10]. This is because of two main reasons: First, the huge internal surfaces of such implants may increase the risk of an IAI. Second, the treatment of infections associated with such types of implants is extremely difficult, as the substantial amount of bony ingrowth complicates the implant removal procedure in the event of high-grade infections. However, the vast internal surface area of such implants, when biofunctionalized, can be used to prevent IAIs.

The surface biofunctionalization of orthopedic implants has received much attention including antibiotic-releasing coatings that have been studied for some time and are shown to reduce infections *in vivo* [11,12,13]. Such coatings are, however, ineffective against antibiotic-resistant bacteria. In fact, the widespread use of such coatings may even trigger the emergence of new antibiotic-resistant strains. Alternative approaches are, therefore, needed to combat IAIs [14].

Inorganic nanoparticles (NPs), such as Ag, are among the most effective antibacterial agents against antibiotic-resistant bacteria, because they exhibit strong antibacterial activity against a wide microbial spectrum with very limited risk of resistance [15]. Clinically, Ag-coated megaprostheses have been shown to reduce infection rates in oncology patients receiving an implant following tumor resection [16,17,18,19]. However, some concerns have been raised regarding the cytotoxicity caused by Ag-coated surfaces [20].

Here, we studied the *in vivo* biocompatibility and infection prevention performance of biofunctionalized AM porous titanium against methicillin-resistant *Staphylococcus aureus* (MRSA). The surface biofunctionalization of such implants is challenging due to the difficulties associated with the homogeneous treatment of their entire surface area and the creation of a durable protective layer. Electrochemical surface modifications have been particularly successful in producing antibacterial surfaces on porous biomaterials [21]. Among them, plasma electrolytic oxidation (PEO) is a one-step process that transforms the native titanium oxide layer into a bioactive surface in a matter of minutes. PEO biofunctionalization with Ag has resulted in implant surfaces with strong antibacterial activity *in vitro* [22,23,24,25]. Furthermore, PEO biofunctionalization has resulted in osteogenic implants *in vivo* [26,27,28]. More recently, we have applied PEO and Ag NPs to produce multifunctional surfaces on AM porous implants [29]. These implants demonstrated antibacterial activity *in vitro* and *ex vivo* against MRSA without inducing any cytotoxicity. However, the antibacterial properties of implants biofunctionalized using PEO with Ag have never been tested *in vivo*.

In this *in vivo* study, we investigated the infection prevention capacity of AM implants biofunctionalized using PEO with Ag NPs by implanting the implants in the intramedullary canal of rat tibiae. We explored the use of bioluminescent bacteria to follow the course of infection in this model. These bacteria are genetically modified to emit light while living. The development of infection in the same animal can, thus, be monitored in real time and noninvasively, thereby increasing the number of time points at which the infection metrics can be measured [30,31,32]. We then analyzed the development of the infections and bone morphology associated with each implant type.

## 2. Materials and Methods

### 2.1. Study Design

Volume-porous Ti-6Al-4V implants were manufactured with selective laser melting (SLM) and were, subsequently, biofunctionalized with PEO using Ag NPs as the active antibacterial agent, resulting in three different types of implants: nontreated implants without any surface modification (NT), PEO-treated implants without Ag NPs (PT), and PEO-treated implants with Ag NPs (PT-Ag). Thereafter, infections were initiated, and the implants were implanted into the intramedullary cavity of the rat tibia. The infections were initiated in two different ways: either through *in vivo* injection of the MRSA bacteria into the intramedullary cavity and the subsequent implantation of the implant or by *in vitro* inoculation of the implant with MRSA prior to implantation into the intramedullary cavity. There are generally two relevant scenarios in implant-associated infections. In the first, the bacteria are not settled on the implant and originate from elsewhere, while the second case concerns bacteria that have already been in contact with the implant for a certain period of time. *In vivo* injection of bacteria mimics the first case. An example of the first case is a clinical situation in which an infection has manifested in the surrounding tissues and the implant should be capable of treating that infection. The *in vitro* inoculation of the implant represents the second situation, for example, in which a nonsterile implant is implanted. Altogether, seven different experimental groups can be identified (Table 1): In the first three groups, the *in vivo* injection of bacteria into the intramedullary cavity was immediately followed by the implantation of the NT implants (inject-NT, *n* = 9), PT-Ag implants (inject-PT-Ag, *n* = 5), or no implants (inject-no-implant, *n* = 3), with the parameter *n* being the number of implants per group. In the 4 remaining groups, the *in vitro* inoculation of the implant prior to the implantation was performed with MRSA bacteria for the first 3 groups, including NT implants (ino-NT, *n* = 6), PT implants (ino-PT, *n* = 6), and PT-Ag implants (ino-PT-Ag, *n* = 6), and without bacteria for the last group, i.e., NT implants (ino-NT-no-infection, *n* = 2). The sample size was estimated based on a previous study with this model [33] in which we deemed a reduction of bacterial levels by 90% to have a clinically relevant effect. Using a power of 80%, the sample size was determined as 6. To clearly demonstrate infection prevention, the number of implants for the inject-NT group was enhanced to 9. To reduce the number of animals, the inject-no-implant and ino-NT-no-infection groups were limited to *n* = 3 and *n* = 2, respectively.

### 2.2. Implant Design and Additive Manufacturing

The rationale behind the implant design has been presented elsewhere [29]. The geometry of the implant was adapted to make it fit the intramedullary tibial rat model. The final design of the implant was 1.1 mm in diameter and 15 mm in length (total). It had a solid proximal part of 3 mm to prevent the leakage of fluids from the intramedullary cavity into the knee joint. The implants were additively manufactured in-house using a selective laser melting (SLM) machine (SLM-125, Realizer, Borchem, Germany) with an LM-400-AC ytterbium laser (IPG Photonics Corporation, Oxford, MI, USA). The laser power was 96 W with a wavelength of 1070 ± 10 nm and an exposure time of 300 µs. The implants were fabricated under an argon flow, resulting in an oxygen content < 0.2%. Medical-grade (grade 23, ELI) Ti-6Al-4V powder (AP&C, Boisbriand, Quebec, Canada) with a spherical particle morphology and particle sizes ranging from 10 to 45 µm was used as the feedstock. Following the SLM, loose powder particles were cleared by vacuum cleaning. The specimens were subsequently ultrasonicated in acetone, 96% ethanol, and demineralized water for 5 min each.

### 2.3. Surface Biofunctionalization

The implant surface was biofunctionalized with PEO using electrolytes consisting of 0.15 M calcium acetate and 0.02 M calcium glycerophosphate (both from Sigma-Aldrich, St. Louis, MI, USA) dissolved in demineralized water. In the case of the PT-Ag implants, 3.0 g/L Ag NPs (Sigma-Aldrich) with a spherical morphology and particles sizes between 7 and 25 nm were dispersed into the PEO electrolytes. The PEO electrolytes were sonicated twice for 3 min, and they were stirred in between at 500 rpm for 5 min with a magnetic stirrer (IKA-Werk GmbH & Co. KG, Staufen, Germany) using a stir bar of 40 × 8 mm (VWR, Radnor, PA, USA).

The PEO biofunctionalization process was performed using a custom-made set-up consisting of an AC power source (50 Hz, ACS1500, ET powder Systems Ltd., Chesterfield, UK), a data acquisition board (SCXI, National Instruments, Austin, TX, USA) that connected the computer interface to the power supply, and two electrodes placed in a double-walled glass electrolytic cell that contained 800 mL of the electrolyte. The implants served as the anode, while a cylindrical-shaped stainless-steel ring placed against the inner wall of the electrolytic cell formed the cathode. The PEO processing was performed with a constant current density of 20 A/dm^2^ for 5 min. Homogeneous particle distribution was ensured through continuous stirring of the electrolyte at 500 rpm. During biofunctionalization, the voltage-time (V-t) transients were recorded every second, and the temperature was kept constant at 5 ± 2 °C using a thermostatic bath (Thermo Haake, Karlsruhe, Germany). The PEO treatment was followed by 1 min of rinsing of the implants in running tap water and autoclaving for sterilization.

### 2.4. Characterization of the Surface Morphology and Chemical Composition

The surface morphology of the implants prior to and after the PEO treatment was characterized using a scanning electron microscope (SEM, JSM-IT100LV, JEOL, Tokyo, Japan). Before imaging, a gold layer of 5 ± 2 nm was sputtered onto the specimens. The chemical composition of the implants was determined using energy-dispersive X-ray spectroscopy (EDS).

### 2.5. Ion Release Kinetics

To investigate the release kinetics of the Ag ions from the PT-Ag implants, 3 specimens from each group were immersed in 1 mL phosphate-buffered saline (PBS) in a dark Eppendorf tube and kept at 37 °C in a water bath under static conditions. Subsequently, the specimens were extracted after 0.5, 1, 2, 4, and 7 days of immersion. The concentration of the elements was then measured using inductively coupled plasma optical emission spectroscopy (ICP-OES) (PerkinElmer Optima 3000DV, PerkinElmer, Zaventem, Belgium).

### 2.6. X-ray Diffraction

The phase composition of the implants was analyzed with X-ray diffraction (XRD) using a D8 advanced diffractometer (Bruker, Billerica, MA, USA). The settings were as follows: voltage = 45 kV, current = 40 mA, scatter screen height = 5 mm, divergence slit = V6, and CuKα radiation detector = LL 0.11 W 0.14. The specimens were analyzed with a coupled *θ*–2*θ* scan ranging between 20 and 120°, a counting rate of 5 s/step, and a step size of 0.030° 2*θ*. Thereafter, the acquired data were analyzed using DiffracSuite.Eva (version 5.0, Bruker).

### 2.7. Preparation of Bacterial Culture and Implant Inoculation

The antibacterial properties of the implants were tested *in vivo* in a rat tibial infection model against the MRSA strain AH4802 [34]. The preparation of the bacterial inoculum was initiated one day prior to surgery by suspending a single colony into 3 mL of tryptic soy broth (TSB) and incubating it overnight at 37 °C. Thereafter, the bacteria were washed and centrifuged twice in PBS at 14,000 rpm for 2 min, and the optical density was measured at a wavelength of 600 nm. For intramedullary injection, the inoculum was diluted to a concentration of 10^6^ CFU/10 µL. For the inoculation of the implant, an inoculum of 10^8^/mL was prepared in Eppendorf tubes, and the implants were incubated statically in the horizontal position for 1 min. To determine the number of CFUs present on the implants after the inoculation process and prior to implantation, the implants (*n* = 3/group) were sonicated in PBS and the inoculum was quantified by plating 10-fold serial dilutions in quadruplicates onto blood agar plates followed by overnight incubation at 37 °C and CFU quantification.

### 2.8. Animal Experiment

The animal experiment was approved by the local ethics committee for animal experiments (Utrecht University, The Netherlands) and the central authority for scientific procedures on animals (approved protocol AVD115002017446). This study was conducted according to the ARRIVE guidelines for reporting animal research [35]. For the experiment, 14-week-old male Sprague–Dawley rats (Charles River, L’Arbresle, France) were housed in groups of three in individually ventilated cages at the central laboratory of the animal institute (Utrecht University). Food and water were available *ad libitum*. The animals were housed in the animal facility one week prior to the experiment to acclimatize and were randomly allocated to an experimental group using the RAND function in Microsoft Excel.

Prior to surgery, the animals were given 0.03 mg/kg of buprenorphine (Temgesic^®^, RB Pharmaceuticals Limited, Slough, United Kingdom) and 4 mg/kg of carprofen (Rymadil^®^, Pfizer Animal Health, Capelle aan den IJssel, The Netherlands) subcutaneously, as well as carprofen post-operatively after 24 h. The surgery was performed under general anesthesia with 2–3% isoflurane. The left hind leg was shaved and soaked in iodine to disinfect the skin. Subsequently, a para-patellar incision was made to open the skin and fascia. The patella tendon was dissected laterally and dislocated medially. Next, a hole was drilled through the cortical bone into the intramedullary canal of the tibia. Infection was induced either through the *in vivo* injection of 10 µL bacterial inoculum into the medullary cavity using a micro syringe (Hamilton, Reno, NV, USA) or through 1 min incubation *in vitro* of the specimens in 1 mL of the prepared bacterial inoculum (static, horizontal position). Thereafter, the implant was inserted into the intramedullary canal. If no implant was inserted, the hole was sealed with bone wax. Subsequently, wound closure was performed using PDS II and Monocryl sutures (both from Ethicon, Somerville, NJ, USA). The rats were euthanized after 7 days with CO_2_.

The surgery was performed by two surgeons in a laminar flow cabinet, assisted by one assistant to guarantee sterile conditions throughout the surgery. In total, 43 animals were used. Based on a previous study using this model [33], we anticipated a bacterial load of 2 × 10^6^ CFU on the NT implants after 1 week with a standard deviation of 60%. A reduction of 90% in the bacterial load was considered clinically significant. Assuming an 80% power, the required sample size was 6 per group. Considering the risk of dropouts, we decided to use 7 animals per group. The inject-NT group included 10 animals to obtain an accurate estimate of the infection rate. Furthermore, the inject-no-implant group had 3 animals, as this was merely a control group for the presence of an implant. In addition, the ino-NT-no-inf group had 2 animals, as it had to simply confirm that we had conducted the surgery under sterile conditions. Because of the misalignment of the implant with the intramedullary canal, 6 specimens were excluded from the analysis, including 1 inject-NT, 2 inject-PT-Ag, 1 ino-NT, 1 ino-PT, and 1 ino-PT-Ag specimens.

### 2.9. Bioluminescence Measurements

On the day of surgery and at 1, 3, 5, and 7 days after surgery, the bioluminescent signal of the bacteria was measured for 5 min using the optical imaging system of MIlabs (Utrecht, The Netherlands) while the animals were under general anesthesia with 2% isoflurane. The bioluminescent images were processed using the optical imaging unit of the MIlabs software (version 2.3.5). A square-shaped region of interest (ROI) with a size of 260 × 260 pixels was used to measure the integrated density of the determinant leg. Subsequently, an ROI of the same size was used to measure the integrated density of the background. Ultimately, the integrated density of the luminescent area was determined by subtracting the background signal from the ROI of the concerned leg.

### 2.10. Micro-CT

Tibiae were harvested and cleansed from their surrounding tissue under sterile conditions. Subsequently, micro-CT scanning was performed with a Quantum FX scanner (PerkinElmer, Waltham, MA, USA) using a tube current of 180 mA, a tube voltage of 90 kV, and a 20 mm field of view. The images were stacked with a resolution of 20 µm and analyzed using the BoneJ plugin (version 1.3.12) of ImageJ (version 1.48).

The implant was excluded from the analysis based on a global threshold. To cover the same bone area in all of the specimens, the proximal fusion point between the tibia and fibula served as an anatomical reference. The total bone volume (BV) was determined for 600 slices distally (1.2 cm) from the point of reference. The bone was segmented by applying an adaptive threshold based on the mean local grayscale distribution. The peri-implant BV was defined as the volume of the bone tissue present in the region of interest (ROI) within the inner cortical perimeter, while the cortical BV represented the BV present outside the ROI. Both the peri-implant and cortical BVs were determined for 10 slides at 3 mm (proximal) and 9 mm (distal) from the point of reference. The porosity of the cortical bone tissue was also determined. In addition, the outer perimeter of the harvested tibiae was measured as a sign of cortical expansion.

### 2.11. Osteomyelitis Score

Bone changes indicating osteomyelitis were scored twice by 2 blinded observers using the raw micro-CT scans with the following criteria: 0 (no abnormalities), 1 (mild osteolysis and/or periosteal response), 2 (significant osteolysis and/or cortical thickening), 3 (focal loss of cortex with extensive osteolysis), and 4 (complete loss of cortical morphology).

### 2.12. CFU Count

To quantify the CFU count, a 1 cm long bone sample was obtained from the proximal part of the tibia with a sterilized saw (Dremel rotary saw, Breda, The Netherlands). Subsequently, the implant and the bony tissue were separated. The bony tissue was weighed and homogenized (Polytron PT3100, Kinetic Benelux, Best, The Netherlands). The implants were rinsed three times in PBS and were subsequently sonicated for 1 min. Thereafter, the implants were weighed, serial dilutions were prepared on blood agar plates, and the number of CFUs was counted after overnight incubation. The CFU count was normalized to the weight of the bones and the full length of the implants to determine the normalized CFU counts of the bone tissue and the implants, respectively. The assessment of the contralateral (i.e., right) tibiae did not demonstrate any bacterial infection in any of the animals.

### 2.13. Biofilm Formation

The capacity of the implants to prevent biofilm formation (*n* = 2/group) was analyzed on the distal part of the implant. Using a sterilized saw (Dremel rotary saw, Breda, The Netherlands), a 0.5 mm thick bone slice was cut. The implant and the bony tissue were subsequently separated. The bony tissue was used for histology. The implant was rinsed twice in PBS and was, subsequently, fixated in 4% paraformaldehyde. Thereafter, the implants were rinsed with demineralized water for 5 min and dehydrated in 50% ethanol for 15 min, in 70% ethanol for 20 min, in 96% ethanol for 20 min, and in hexamethyldisilane for 15 min. Finally, the implants were air-dried for 2 h and inspected using SEM.

### 2.14. Histology

For the histological analysis, the bone surrounding the distal part of the implant was fixated in 4% paraformaldehyde. Thereafter, the bone specimens were decalcified in 0.3 M EDTA, embedded in paraffin, and cut into 6 µm thick sections using a sawing microtome (Leica, Nussloch, Germany). The slices were stained using H&E staining and imaged with a brightfield microscope (DM500, Leica, Nussloch, Germany).

### 2.15. Statistical Analysis

All data are expressed as mean ± standard deviation. The statistical analyses were performed with GraphPad Prism (GraphPad Software, version 9.3.0, La Jolla, CA, USA) using one- and two-way ANOVA followed by a Bonferroni *post hoc* test. The differences between the groups were considered statistically significant when *p* < 0.05.

## 3. Results

### 3.1. Implant Synthesis and Surface Biofunctionalization

**Figure 1:**
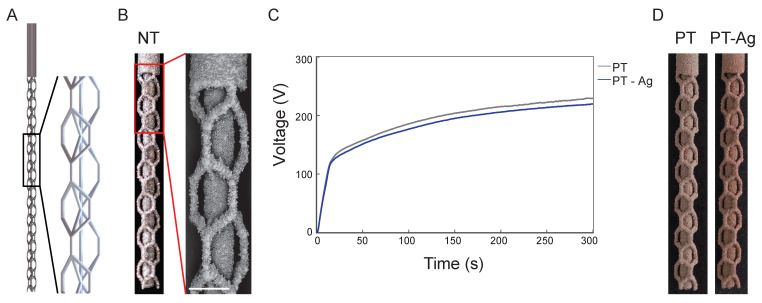
The design, surface morphology, and V-t transients of the AM porous titanium implants: (**A**) design and (**B**) macroscopic, as well as microscopic, images of the AM porous implants designed using a repetitive unit cell with a diameter of 0.5 mm and a solid and porous part of 3 and 12 mm length, respectively. The middle strut in (**A**) is depicted relatively thinner to clearly show the design of the implant. (**C**) The V-t transients recorded during the surface biofunctionalization of the PT and PT-Ag implants using PEO. (**D**) The macroscopic images of the PT and PT-Ag implants after the PEO biofunctionalization process. Scale bar = 500 µm.

The 3D implant design with a repetitive unit cell structure is presented in Figure 1A. The implants synthesized using SLM displayed a highly porous structure with partially molten particles attached to the implant surface (Figure 1B). The V-t transients of the PEO process demonstrated similar characteristics for the PT and PT-Ag implants (Figure 1C). At the initial stage, the voltage rose sharply to 93 ± 3 V after 10 s until dielectric breakdown occurred, followed by a gradual increase in the voltage until a final voltage of 220 ± 5 V and 229 ± 4 V was reached for the PT-Ag and PT implants, respectively. The PEO processing markedly altered the macroscopic appearance of the implants (Figure 1D).

### 3.2. Biomaterial Characterization

**Figure 2:**
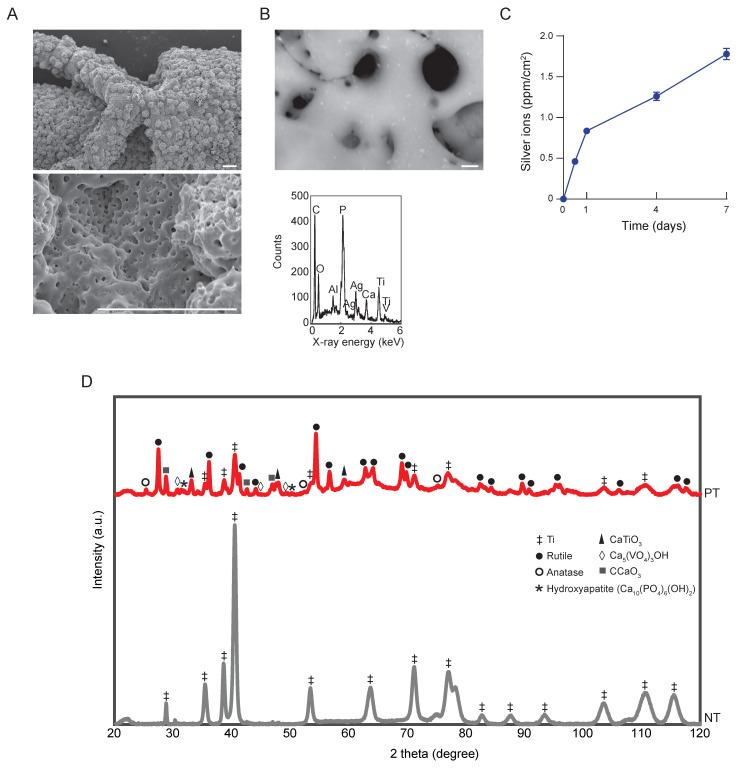
The surface characterization of the PEO-biofunctionalized implants: (**A**) SEM imaging of the surface of the PT implants, scale bar = 100 µm; (**B**) EDS analysis of the PT-Ag implant surfaces with EDS, scale bar = 1 µm; (**C**) Ag ion release kinetics of the PT-Ag implants (*n* = 3) over 7 days as measured using ICP-OES; (**D**) X-ray diffraction spectra of the NT and PT implants.

The SEM imaging demonstrated a highly porous surface with interconnected pores, which homogeneously spanned the entire surface of the PEO-biofunctionalized implants (Figure 2A). The EDS analysis confirmed the presence of Ag NPs (Figure 2B). Ti, Al, and V were detected as components of the implants. Ca, P, O, and C, which were present in the PEO electrolyte, were also detected. Ag ions were continuously released from the PT-Ag implants, resulting in a cumulative ion release of 1.83 ± 0.06 ppm/cm^2^ after 7 days (Figure 2C). The XRD analysis demonstrated that the phase composition of the NT implants consisted entirely of the Ti phase, while this phase was converted into rutile, as well as anatase, TiO_2_ phases on the PT implants (Figure 2D). Furthermore, CaTiO_3_, Ca_5_(VO_4_)_3_OH, CCaO_3_, and hydroxyapatite were detected on the surface of the PT implants. The phase composition of the PT-Ag implants was identical to that of the PT implants and is, therefore, not presented.

### 3.3. Antibacterial Properties

**Figure 3:**
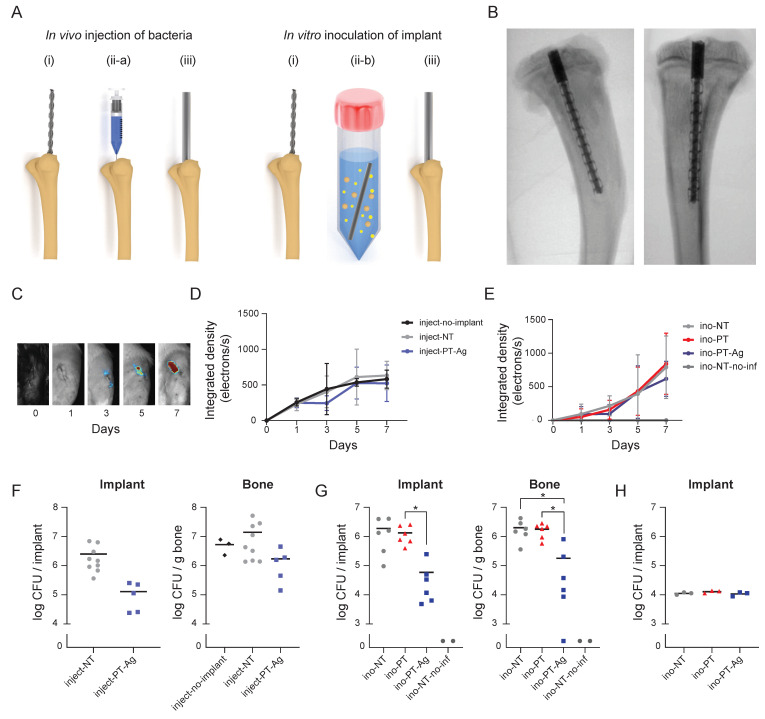
Antibacterial activity of the implants against MRSA. (**A**) Both methods of initiating the infection: *in vivo* intramedullary injection of bacteria into the intramedullary cavity (left) and *in vitro* inoculation of the implant prior to implantation (right). The surgical procedure consisted of (i) drilling a hole through the tibial plateau followed by either (ii-a) *in vivo* injection of bacteria or (ii-b) 1 min *in vitro* inoculation of the implant and (iii) the implantation of the implant into the intramedullary canal. (**B**) The micro-CT images of the implant in the tibia from medial (left) and posterior (right) positions. (**C**) The bioluminescence images and spectra of the bioluminescent MRSA AH4802 *in vivo* over 7 days in the intramedullary cavity for the infections initiated through (**D**) *in vivo* intramedullary injection or (**E**) through *in vitro* inoculation prior to implantation. The quantification of the bacterial load after 7 days (**F**) after the *in vivo* injection of the bacteria and (**G**) after *in vitro* inoculation on the implant prior to the implantation. (**H**) CFU count following the *in vitro* inoculation of the implants and before their implantation into the tibia. *n* = 10 for inject-NT, *n* = 7 for all other implants, *n* = 3 for inject-no-implant, and *n* = 2 for ino-NT-no-inf. * *p* < 0.05.

**Figure 4:**
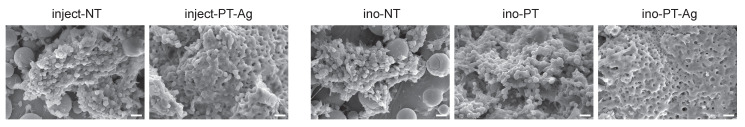
Biofilm formation on the implants visualized using SEM after 7 days. Scale bar = 5 µm.

Infection was initiated either through the *in vivo* injection of bacteria into the intramedullary canal (inject-implants) or the *in vitro* inoculation of the implant prior to implantation (ino-implants) (Figure 3A). After 7 days, the implants in the tibia were visualized using micro-CT (Figure 3B). Bioluminescence imaging (Figure 3C) demonstrated the bioluminescent signal for all of the groups at all time points, except for the ino-NT-no-infection group and day 0 for all of the inject-implants (Figure 3D) and ino-implants (Figure 3E). No significant differences were observed between the groups at any time point. The quantification of the number of CFUs after 7 days on the implants and in the peri-implant bone indicated that there were no differences between the groups in which the bacteria were injected into the intramedullary canal *in vivo* (Figure 3F), although the bacterial load on inject-PT-Ag implants was nearly significantly lower compared to inject-NT implants (*p* = 0.0576). For the groups in which the infection was induced through the *in vitro* inoculation of the implants, the number of the CFUs associated with ino-PT-Ag implants was significantly lower than that of the ino-NT implants (*p* < 0.05). Furthermore, the CFU count of the bony tissue surrounding the ino-PT-Ag implants was significantly lower compared to the ino-NT and ino-PT implants (*p* < 0.05; Figure 3G). The number of CFUs on the ino-implants prior to the implantation did not differ following bacterial inoculation (Figure 3H). The SEM imaging demonstrated biofilm formation on the NT and PT implants, with bacterial cells stacked on top of each other in multiple layers, while there were no signs of biofilm formation on the PT-Ag implants but only a few individual bacteria (Figure 4).

### 3.4. Bone Changes

**Figure 5:**
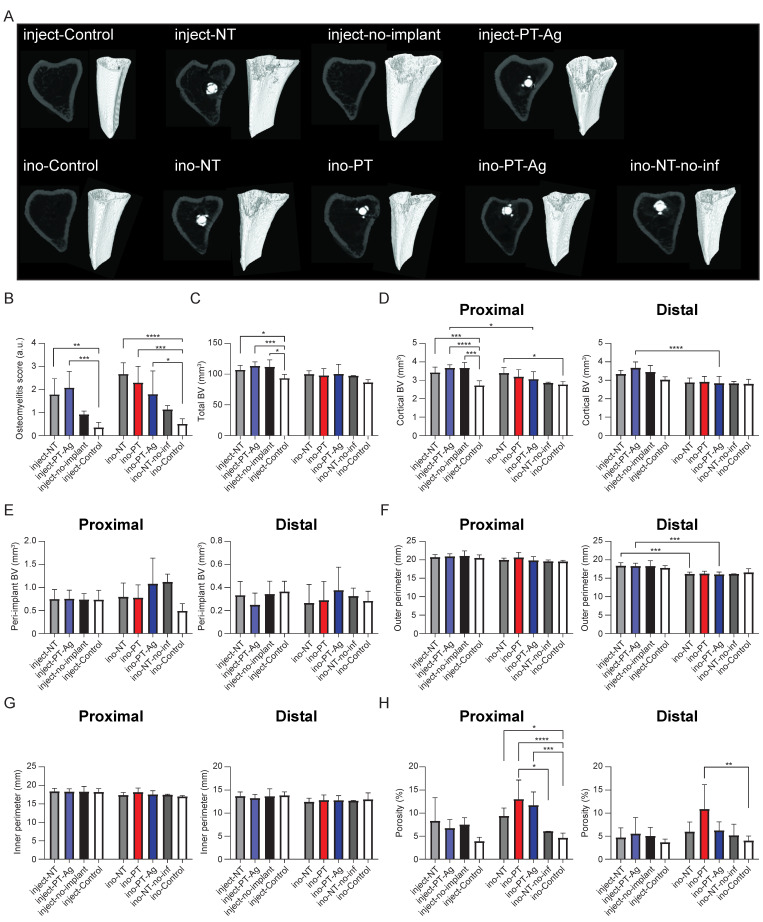
Changes in bone morphology after 7 days: (**A**) micro-CT images of the rat tibiae; (**B**) radiographic osteomyelitis scores. The quantification of several bone morphometric parameters using micro-CT: (**C**) total BV; (**D**) cortical BV; (**E**) peri-implant BV; (**F**) outer perimeter; (**G**) inner perimeter; (**H**) porosity. * *p* < 0.05, ** *p* < 0.01, *** *p* < 0.001, and **** *p* < 0.0001. BV = bone volume.

The micro-CT analysis demonstrated osteomyelitis on infected tibiae as indicated by osteolysis and cortical thickening (Figure 5A). The radiological scoring of the osteomyelitis indicated that the inject-NT (*p* < 0.01), inject-PT-Ag (*p* < 0.001), ino-NT (*p* < 0.0001), ino-PT (*p* < 0.001), and ino-PT-Ag (*p* < 0.05) groups had significantly higher osteomyelitis scores compared to the control group, while the osteomyelitis scores of the inject-no-implant and ino-NT-no-infection groups were not significantly different from that of the control group (Figure 5B). The total BV of the infected left tibia of the inject-NT (*p* < 0.05), inject-PT-Ag (*p* < 0.001), and inject-no-implant (*p* < 0.01) groups were different from that of the inject-control group (i.e., right tibia), while no significant differences were observed between the groups in which the implants were inoculated *in vitro* (Figure 5C). The cortical BV and peri-implant BV were determined both proximally and distally to indicate the location of the changes in the bone morphology. The proximal cortical BV was enhanced for the inject-NT (*p* < 0.001), inject-PT-Ag (*p* < 0.0001), and inject-no-implant (*p* < 0.001) groups compared to the inject-control group. The same held for the ino-NT group compared to the ino-control (*p* < 0.05) group, and for the inject-PT-Ag group compared to the ino-PT-Ag group both proximally (*p* < 0.05) and distally (*p* < 0.0001; Figure 5D). The peri-implant BV did not differ significantly between the groups (Figure 5E). The outer perimeter was significantly increased for the inject-NT group compared to the ino-NT group (*p* < 0.001). The same observation was made for the inject-PT-Ag group compared to the ino-PT-Ag group (*p* < 0.001; Figure 5F), while the inner perimeter was similar among all groups (Figure 5G). The cortical bone porosity of the *in vivo* injection groups did not differ, while it was significantly enhanced proximally for the ino-NT (*p* < 0.05), ino-PT (*p* < 0.0001), and ino-PT-Ag (*p* < 0.001) groups compared to the ino-control group. The same observation was made for the ino-PT group compared to the ino-NT-no-infection (*p* < 0.05) group and distally for the ino-PT group compared to the ino-control group (*p* < 0.01; Figure 5H).

### 3.5. Histology

**Figure 6:**
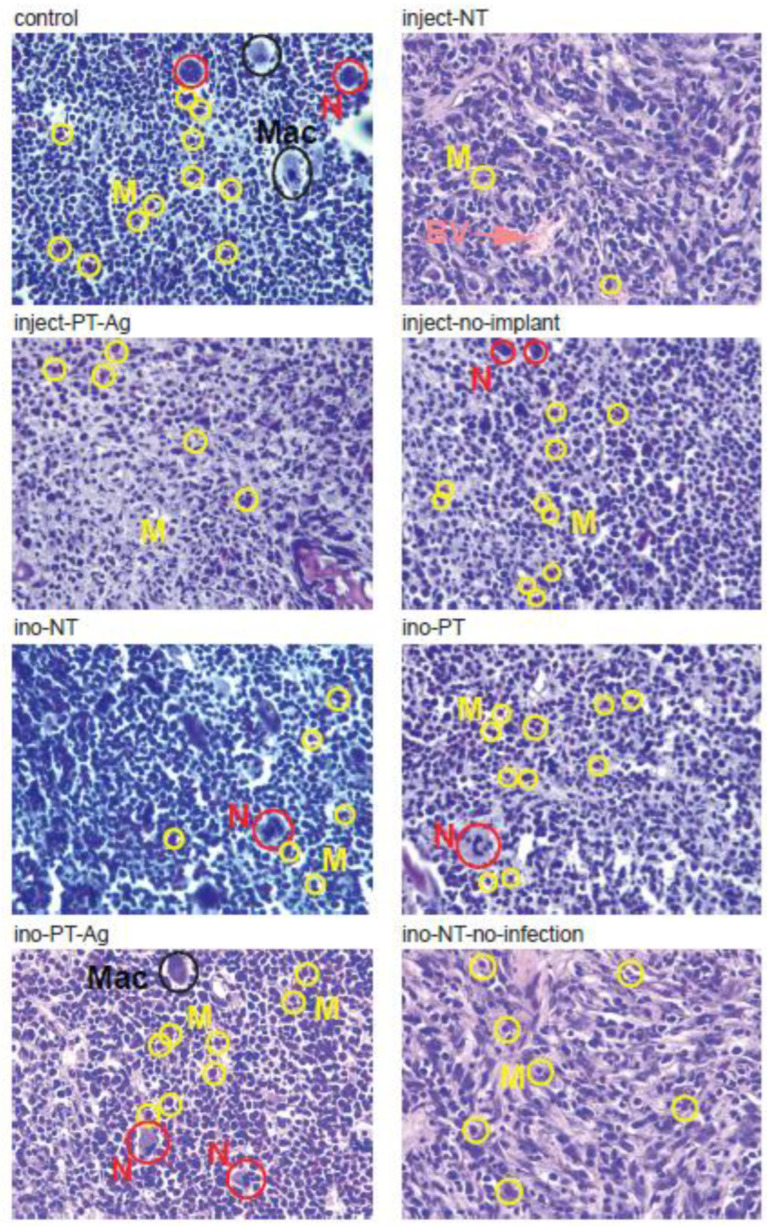
The histology sections of the rat tibia 7 days after surgery. The H&E staining of the rat-tibia indicating the presence of neutrophils (red circles), monocytes (yellow circles), macrophages (black circles), and blood vessels (pink arrow). The images were taken with 40× magnification. N = neutrophils; M = monocytes; Mac = macrophages; BV = blood vessel.

The bone marrow surrounding the implants was visualized with H&E staining (Figure 6). The immune cells were found to have infiltrated the bone marrow surrounding the implants with monocytes present in all of the tissues sections, while neutrophils were present in most and macrophages in some of the sections. The types of the immune cells identified were similar among the different treatment groups.

## 4. Discussion

Implants with intrinsic antibacterial properties are urgently needed to prevent IAIs, thereby increasing the longevity of orthopedic implants. Over the last few years, AM porous titanium implants biofunctionalized using PEO have demonstrated promising results in this direction. However, the antibacterial properties of such implants have, thus far, not been assessed *in vivo* [36].

Here, we used a rat implant infection model to evaluate the *in vivo* biocompatibility and infection prevention performance of AM titanium biofunctionalized using PEO with Ag NPs. We observed that the PT-Ag implants reduced the bacterial load compared to the NT and PT implants. Furthermore, the method used for inducing the infection affected the course of infection and the relative performance of the different experimental groups.

### 4.1. In Vivo Implant Infection Models: Prevention vs. Treatment and the Role of the Inoculation Method

It is important to note that the PEO-biofunctionalized implants are primarily designed for infection prevention and not infection treatment. An implant infection model that faithfully represents the actual clinical conditions, with low infection rates, would need a prohibitively large number of animals, rendering such an experiment infeasible due to the practical, ethical, and financial considerations. Researchers have, therefore, explored alternative approaches to mimic the clinical situation as closely as possible while limiting the required number of animals. While there are late-onset infections caused by hematological pathogens, the majority of IAI cases arise during the first 3 weeks after surgery and are caused by bacteria entering the wound area [37]. While the number of bacteria entering the wound peri-operatively is generally limited [38], a much higher bacterial load should be used in animal experiments to demonstrate an antibacterial effect using a limited number of animals. The biofunctionalized implants would then have to demonstrate antibacterial activity against a much higher bacterial load than is needed in clinical settings [39]. That would translate to a higher required dose of the antibacterial agent, which may increase the risk of cytotoxicity [40].

IAIs can be initiated by the bacteria present on improperly sterilized implants [41] or by those reaching the wound area through the surgeon’s hands [42], migrating from the tissues adjacent to the wound area [43], or, in the case of late-onset IAIs, by pathogens originating from the bloodstream [44]. As we were primarily interested in the prevention of early-onset IAI, we investigated the first two methods of infection—either through the *in vivo* injection of bacteria into the intramedullary canal or by the *in vitro* inoculation of implants with bacteria prior to implantation—and we followed the course of infection for up to 7 days.

The *in vivo* injection of bacteria into the intramedullary cavity has the disadvantages that a very high bacterial load is already present inside the bone before the implant is inserted and that bacteria may infect the bony tissue rather than adhering onto the implant surface. As a result, the implant may need to possess a strong antibacterial activity to clear bacteria from the infected tissue. This model, therefore, mimics a clinical situation in which a (severe) infection is already present in the surrounding tissues, and the implant should be capable of treating that infection. Here, we did not observe a difference in the bacterial load after 1 week between inject-no implant, inject-NT, and inject-PT-Ag in the bone tissue, while the bacterial load on the implant was nearly significantly reduced for the inject-PT-Ag implants compared to the inject-NT implants. The comparison between the inject-no implant and inject-NT is particularly important in this regard. It is known that the presence of an implant frustrates the immune system, thereby decreasing the required infection dose by up to 10^6^-fold [45]. It is, therefore, expected that infection clearance is less effective in the inject-NT group compared to the inject-no-implant group. The fact that there were no significant differences between these two groups suggests that the course of infection is primarily driven by the bacteria infecting the surrounding tissues upon injection and not the presence of the implant.

The *in vitro* inoculation of the implant mimics a situation in which an unsterile implant is implanted. This situation is suitable for the study of the prevention of IAIs, as the bacteria are in the vicinity of the implant and are, thus, more likely to proliferate on or near the implant surface than in the bony tissue. The results of this study support this hypothesis, as the ino-PT-Ag implants reduced the bacterial load in the bony tissue by 90% compared to the ino-NT and ino-PT implants, as well as the bacterial load on the implant for the ino-PT-Ag implants compared to the ino-PT implants.

There were no differences between ino-PT and ino-NT, indicating that the PEO biofunctionalization process does not increase the risk of infection. Furthermore, ino-NT-no-infection had no bacteria, showing that the surgery had, indeed, taken place under sterile conditions. In addition, all controls (i.e., right tibiae) were culture-negative, indicating that the infection was localized at the site of contamination.

In addition to the way the infection is initiated, the animal species affects the outcome of the experiment. Rat models are versatile and low cost, making them appropriate for screening before preclinical tests using full-sized implants are performed in larger animals [46]. However, rats have a strong immune system, which requires a high bacterial load compared to, for example, rabbits, which are very sensitive to infection [47]. On the other hand, mice are much smaller, rendering both the surgery and the fabrication of the implants excessively challenging.

We implanted the implant intramedullary into the tibia, as this more closely resembles the clinical situation compared to subcutaneous implantation. The local environment does play a role in the infection as different types of immune cells may be present at different anatomical sites. Moreover, the tissue microenvironments are different, and the cytotoxicity levels differ between the bony and skin tissue, making it difficult to study the specific aspects that are relevant for bone-related infections [48]. Furthermore, the intramedullary insertion of the implant mimics the implantation of an implant in orthopedic patients.

### 4.2. Bioluminescence Imaging

In this study, we used bioluminescence imaging to track the course of infection in real time. We continued to receive the bioluminescent signal at all time points, except for the ino-NT-no-infection group and at day 0 for all of the groups. While we observed no differences in the bioluminescent signal among the different groups, we detected differences in the bacterial load on day 7 in terms of the CFU count. The strength of the bioluminescent signal and the resolution of the scanner are likely not sensitive enough to pick up these differences in the bacterial load within the reduction range found [32,49], as there is still a number of bacteria within the studied region of interest and, thus, a bioluminescence saturation effect might have been reached. Nevertheless, we did detect an increase in the bioluminescence signal from day 0 onwards. Further optimization should make it possible to use bioluminescent bacteria as a powerful tool to track the course of infection in this intramedullary tibial infection model, as it enhances the number of data points and is likely to reduce the number of required animals.

### 4.3. Surface Biofunctionalization of AM Porous Implants

The implants developed in this study were volume-porous implants produced using AM. Previous research has indicated that highly porous materials are more prone to infection compared to fully dense materials, although this difference disappears when the implant is overgrown with the surrounding tissue [50]. It is important to assess the infection risk of highly porous AM implants, since the use of AM is expected to increase vastly because of the customization opportunities offered by free-form fabrication and the possibility to optimize the mechanical properties of such geometrically ordered porous implants [51]. The design objective often is to enhance the bony ingrowth [52,53] while reducing the risk of IAI [54].

The surface biofunctionalization of porous implants is challenging. PEO has been utilized frequently to generate multifunctional implants that possess both antibacterial and osteogenic properties [55,56,57,58]. While the antibacterial properties have been evaluated extensively *in vitro*, the antibacterial properties of implants biofunctionalized using PEO have, thus far, not been assessed *in vivo*. However, the osteogenic properties have been analyzed in various animal models and have resulted in (i) enhanced osseointegration and push-out bonding strength in the femora of a rabbit model after 12 weeks [59], (ii) strong bone matrix deposition and enhanced bone-to-implant contact in pig mandibles after 8 weeks [26], and (iii) shortened osseointegration time, increased bone mineral deposition, and enhanced bone–implant contact in rabbit mandibles over 12 weeks [27]. On the other hand, the use of Ag on titanium implants has been shown to result in potent antibacterial implants *in vivo* as evidenced by the prevention of infections caused by *S. epidermidis* in a murine tissue cage model [60], a reduction in bacterial loads when the implants were implanted into rat femora [61], and a 2-log reduction in the bacterial load in a rabbit tibial infection model [62].

Apart from the antibacterial activity, there are some concerns regarding cytotoxicity of Ag-based surface treatments, as Ag is both more antibacterial yet also more cytotoxic compared to other antibacterial agents, such as Cu and Zn [40]. Therefore, the key is to find the balance between the antibacterial activity and osteogenic activity. The toxicity of Ag NPs depends on their size and subsequent Ag ion release [63]. Furthermore, low doses of Ag NPs have been found to support bone fracture healing *in vivo* [64] and are demonstrated to enhance bone regeneration, especially when combined with Ca/P or hydroxyapatite-containing surface layers [65,66]. In this regard, it is important to stress that Ag is more likely to be suitable for infection prevention rather than treatment, since treatment would require high Ag doses.

### 4.4. Bone Morphology and Immune Response

Apart from the CFU count, we studied the changes in bone morphology after 7 days, as the inflammatory reaction to *S. aureus* infection is characterized by dynamic bone changes resulting in quiescent, resorbed, and new bone [67]. Novel bone formation precedes cortical osteolysis [68,69], since it is a direct response to the inflammatory environment, while cortical osteolysis is partially initiated by bacterial presence in the cortical Haversian and Volkmann canals [70]. This can be monitored by scoring the radiographic appearance on micro-CT images. This scoring is capable of distinguishing between infected and noninfected bone, but there is no direct relationship between the bacterial load and the radiographic scores. This is because bone requires several weeks to remodel back to its native architecture [71], and the bone remodeling caused by infection has been shown to be strain-dependent due to the different immune responses they elicit [72,73] and the varying levels of the secretion of toxins that modulate bone regeneration [74].

We observed an increased osteomyelitis score among all of the infected groups compared to the control conditions (Figure 5B). Furthermore, the total BV and proximal cortical BV (Figure 5C,D) were enhanced for all infected inject-implants compared to inject-control, while bone porosity was enhanced for all infected ino-implants compared to the ino-control group, as well as for the ino-PT group compared to the ino-NT-no-inf group. Moreover, there were no differences in the bone morphology between the ino-NT-no-inf and ino-control groups, indicating that the observed changes in bone morphology were due to the presence of infection and not due to the surgical procedure. In addition, the proximal cortical BV (Figure 5D) and distal outer perimeter (Figure 5F) were enhanced for the inject-PT-Ag group compared to the ino-PT-Ag group, indicating that the PT-Ag implants were less able to prevent bone changes when the infection was initiated through *in vivo* intramedullary injection compared to the *in vitro* inoculation of the implant prior to implantation. While the PT-Ag implants reduced the bacterial load, they did not eradicate all the signs of infection, including bone morphology changes. To achieve this, the infection should have been completely eradicated for several weeks to allow the bony tissue to regenerate [75].

The analysis of the immune cells present in the bone marrow surrounding the implant showed the presence of neutrophils, monocytes, and macrophages. We did not observe clear differences among the experimental groups. In the first week of IAI, the primary immune cells responding to the infection were neutrophils and monocytes, as observed in our histological sections [76].

### 4.5. Future Work

While this work investigated, for the first time, the effects of different modes of infection on the efficacy of additively manufactured implants biofunctionalized using plasma electrolytic oxidation and Ag nanoparticles, there are some limitations that need to be addressed in future studies. Most importantly, the translation to clinical settings would require animal models with immune systems that better represent the human immune system. Moreover, the duration of the experiments should be increased to study the effects of such implants on later stages of implant-associated infections. Finally, the effects of combining biofunctionalized implants with different types of systemic antibiotic therapies on the prevention and/or treatment of infections should also be investigated. The potential of AM porous titanium implants with Ag NPs can be further improved by instigating osteogenic properties and enhancing their antibacterial activity through the addition of other inorganic NPs, such as Cu, Zn, and Sr, that give rise to synergistic antibacterial behavior while also enhancing bone regeneration [25,55,58]. Furthermore, the long-term antibacterial properties of these implants need to be investigated, particularly because silver-biofunctionalized implants have shown some promise in terms of long-term antibacterial properties [65]. Finally, the *in vivo* evaluation of the different variants of PEO-biofunctionalized AM porous titanium implants is limited [36], and it needs to be continued in (large) animal models and clinical trials before translation to clinical settings is possible.

## 5. Conclusions

In this *in vivo* study, we investigated the infection prevention capacity of AM implants biofunctionalized using PEO with Ag by implanting the implants in the intramedullary canal of rat tibia. Bioluminescence imaging showed no significant differences among the experimental groups. In the groups in which the infections were induced through *in vivo* intramedullary injection of bacteria, the presence of the implant did not affect the course of infection. This suggests that this model is more suitable for assessing infection treatment rather than evaluating the infection prevention performance of the implants. When infections were induced through the *in vitro* inoculation of the implants prior to implantation, the bacterial load on the PT-Ag implants was significantly lower compared to the PT implants. Furthermore, the CFU count of the bony tissue surrounding the PT-Ag implants was significantly smaller (90–95% reduction) than those of the tissue specimens associated with the NT and PT implants (day 7). The osteomyelitis scores were enhanced for all of the infected implants compared to the noninfected controls, while the immune response did not differ among the groups. Taken together, the results of this study warrant further preclinical and clinical studies on PEO-biofunctionalized AM implants.

## Figures and Tables

**Table 1:** The experimental groups used in this study.

Bacterial Inoculation Method	Bacterial Infection	Implant	PEO Treatment (PT)	Ag NPs	Label
*In vivo* injection of bacteria into intramedullary cavity	Yes	Yes	-	-	inject-NT (no treatment)
	Yes	Yes	Yes	Yes	inject-PT-Ag
	Yes	-	-	-	inject-no-implant
*In vitro* inoculation of implant prior to implantation	Yes	Yes	-	-	ino-NT
	Yes	Yes	Yes	-	ino-PT
	Yes	Yes	Yes	Yes	ino-PT-Ag
	No (PBS)	Yes	-	-	ino-NT-no-inf

## Data Availability

The raw data required to reproduce these findings are available to download from (https://data.mendeley.com/drafts/7t5g8tp75x). The processed data required to reproduce these findings are available to download from (https://data.mendeley.com/drafts/7t5g8tp75x).
